# Cross-sectional survey of SARS-CoV-2 testing at US airports and one health department’s proactive management of travelers

**DOI:** 10.1186/s40794-022-00164-8

**Published:** 2022-03-20

**Authors:** Anna Shaum, Argelia Figueroa, Danica Lee, Allison Ertl, Erin Rothney, Denise Borntrager, Emily Davenport, Reena K. Gulati, Clive M. Brown

**Affiliations:** 1 Division of Global Migration and Quarantine, Centers for Disease Control and Prevention, 1600 Clifton Road, Atlanta, GA 30329 USA; 2 Division of Public Health Investigations, Denver Department of Public Health and Environment, 101 W Colfax Ave, Denver, CO 80202 USA; 3 Current affiliation: Bill & Melinda Gates Foundation, 500 Fifth Ave. N., Seattle, WA 98109 USA

**Keywords:** SARS-CoV-2, COVID-19, Airports, Air travel, Management

## Abstract

**Background:**

Many health departments and private enterprises began offering SARS-CoV-2 testing to travelers at US airports in 2020. Persons with positive SARS-CoV-2 test results who have planned upcoming travel may be subject to US federal public health travel restrictions.

We assessed availability of testing for SARS-CoV-2 at major US airports. We then describe the management of cases and close contacts at Denver International Airport’s testing site.

**Methods:**

We selected 100 US airports. Online surveys were conducted during November–December 2020 and assessed availability of testing for air travelers, flight crew, and airport employees. Respondents included health department (HD) staff or airport directors.

We analyzed testing data and management practices for persons who tested positive and their close contacts at one airport (Denver International) from 12/21/2020 to 3/31/2021.

**Results:**

Among the 100 selected airports, we received information on 77 airports; 38 (49%) had a testing site and several more planned to offer one (*N* = 7; 9%). Most sites began testing in the fall of 2020. The most frequently offered tests were RT-PCR or other NAAT tests (*N* = 28).

Denver International Airport offered voluntary SARS-CoV-2 testing. Fifty-four people had positive results among 5724 tests conducted from 12/21/2020 to 3/31/2021 for a total positivity of < 1%. Of these, 15 were travelers with imminent flights. The Denver HD issued an order requiring the testing site to immediately report cases and notify airlines to cancel upcoming flight itineraries for infected travelers and their traveling close contacts, minimizing the use of federal travel restrictions.

**Conclusions:**

As of December 2020, nearly half of surveyed US airports had SARS-CoV-2 testing sites. Such large-scale adoption of airport testing for a communicable disease is unprecedented and presents new challenges for travelers, airlines, airports, and public health authorities. This assessment was completed before the US and other countries began enforcing entry testing requirements; testing at airports will likely increase as travel demand returns and test requirements for travel evolve.

Lessons from Denver demonstrate how HDs can play a key role in engaging airport testing sites to ensure people who test positive for SARS-CoV-2 immediately before travel do not travel on commercial aircraft.

## Background

Many health departments and private enterprises, including airlines and airports, began offering SARS-CoV-2 (the virus that causes COVID-19) testing to departing or arriving travelers at US airports in 2020. Testing at several airports began shortly after certain jurisdictions enacted policies in mid-2020 requiring travelers to provide testing documentation [1–3]. The Centers for Disease Control and Prevention (CDC) currently recommends unvaccinated persons get tested with a SARS-CoV-2 viral test (nucleic acid amplification test [NAAT] or antigen test) one to three days before travel in addition to other precautions to minimize transmission risk during travel [4].

Despite general awareness of testing availability and jurisdiction-specific entry testing requirements, there was a need for CDC to have a more thorough understanding of SARS-CoV-2 testing offered at US airports. Understanding the scope of testing at airports is important, as it enables accurate planning with partners for persons who test positive on airport grounds and facilitates targeted messaging for persons who may be tested prior to travel, including what to prepare for in case someone in their travel party tests positive. Additionally, persons with COVID-19 and their close contacts with planned upcoming travel may be subject to federal public health travel restrictions if certain criteria are met [5]. Such restrictions include addition to the public health Do Not Board (DNB) list that prevents travel on commercial flights that arrive, travel within, or depart from the United States of persons infectious with or at risk of becoming infectious with a communicable disease that threatens the public health.

Federal public health travel restrictions are an important tool for reducing risk of transmission during travel and reducing translocation of disease in globally mobile populations. While placing people on the DNB list can prevent those with COVID-19 and their close contacts from traveling by commercial aircraft, use of these authorities requires substantial coordination between CDC; the Department of Homeland Security; state, local, or territorial health departments (S/LTHDs); and airlines. Additionally, testing at airports can add complexity to the management of those who test positive, including the need for travelers with immediate upcoming flights to delay or re-schedule travel in a compressed time frame, need to identify appropriate safe transportation and, in some cases, housing for infected travelers and their exposed close contacts, and challenges in communication between testing facilities and health departments.

CDC’s Division of Global Migration and Quarantine conducted an assessment to determine which US airports were offering SARS-CoV-2 testing to travelers. If testing was offered, we sought to understand when testing began, where testing was offered, who provided the testing, what type of tests were offered, and who was being tested.

We also collaborated with one health department, the Denver Department of Public Health and Environment (Denver HD), to describe their management of travelers testing positive at Denver International Airport, and their traveling close contacts several months after the testing site had been operational. We chose to highlight Denver HD’s process as we were unaware of other health departments who had implemented similar procedures at that time.

## Methods

We conducted a cross-sectional survey of SARS-CoV-2 testing offered at US airports. We selected 100 commercial US airports across 46 jurisdictions (states, territories, and large cities) based on the following pre-determined criteria: 1) having more than 1000 international passenger flight arrivals in 2019, 2) having a CDC quarantine station (QS) on site, or if not meeting one of the above criteria, 3) ranking among the top airports for domestic passenger arrival volume in 2019. These 100 selected airports received a majority of domestic and international air travelers in 2019, representing 84% of passenger volume in the US [6].

The survey assessed information including the availability of testing at the airport, type of test offered and to whom, location of testing site, and date testing began. Staff at the 20 QSs across the US emailed their contacts at S/LTHDs, requesting they identify a person who could complete the survey for selected airports located within their geographic jurisdiction. The survey was coordinated through QS because of the established relationships between QSs and HDs in their jurisdictions [7]. S/LTHD staff had the option to provide a respondent at the health department or to direct the QS to a potential respondent at the airport. If the S/LTHDs did not provide information for a respondent within 10 days or did not reply after three follow-up attempts, then QS staff contacted airport directors inviting them to respond to the survey. The survey opened in late November 2020 and closed after approximately 3 weeks.

Completing the survey was voluntary with informed consent. The survey was administered online with an option to complete by phone if requested. The survey allowed responses for more than one airport if a health department had jurisdiction over more than one airport.

Additionally, we documented the implementation of testing at the Denver airport. The Denver HD provided deidentified data resulting from onsite testing at Denver International Airport from December 21, 2020 through March 31, 2021. This included information on the number of tests conducted, number of positive results, number of persons with positive tests who had imminent travel plans, and number of DNB determinations associated with the testing site. Information on Denver HD’s system for managing travelers was described using the public health order issued to the testing site [Denver Department of Public Health and Environment, unpublished order, 2020] and templates of the isolation and quarantine orders issued to persons who tested positive and their close contact travel companions.

We used R (version 1.2.5033) to analyze the survey data descriptively (counts and percentages). This activity was reviewed by CDC and was conducted consistent with applicable federal law and CDC policy.^1^

## Results

### Airport testing survey

**Fig. 1:**
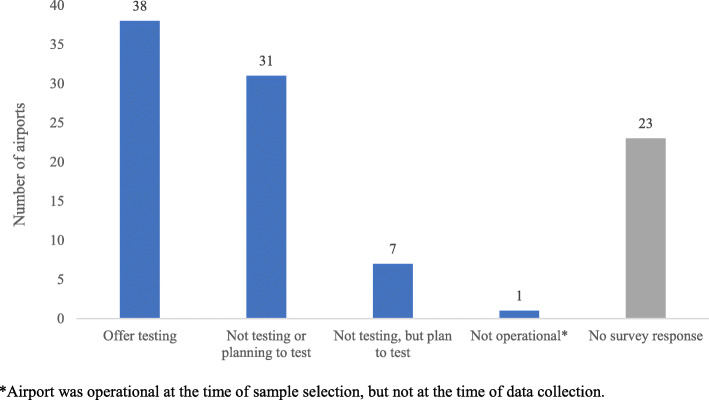
SARS-CoV-2 Testing Availability at Airports, Centers for Disease Control and Prevention Survey, November–December 2020 (*N* = 100)

We received responses from 71 HD or airport employees; one person subsequently declined to participate. The 70 survey respondents represented 77 airports: six provided information on more than one airport (five respondents answered for two airports and one respondent answered for three airports). Most of the surveys were conducted online with airport (*N* = 46; 66%) or HD contacts (*N* = 23; 33%); one (1%) survey was conducted over the phone with a HD contact. Of the 77 airports for which information was obtained, 38 (49%) had an onsite (on airport grounds) testing site or planned to offer one (*N* = 7; 9%). Onsite testing was not offered nor planned at 31 (40%) airports, and one airport was not operational at the time of the survey (Fig. 1).

Of the 38 airports with onsite COVID-19 testing, 30 (79%) offered testing to travelers, and eight (21%) offered testing to non-travelers (e.g., airport or airline crew) only. Seven airports were designated as community testing sites and were open to the general public, including both travelers and non-travelers.

**Fig. 2:**
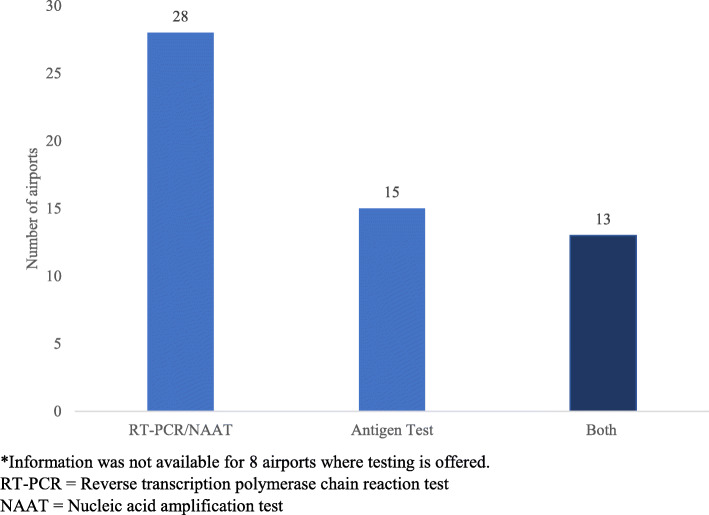
SARS-CoV-2 Test Type at US Airports, Centers for Disease Control and Prevention Survey, November–December 2020 (*N* = 30)*. *Information was not available for 8 airports where testing is offered. RT-PCR = Reverse transcription polymerase chain reaction tes. NAAT = Nucleic acid amplification test

Information on the airport’s testing site location was available for 32 airports. Of those, 18 (56%) were located outside the secure area, and 12 (38%) were located inside the secure area. Five airports had multiple testing sites on airport property, including two (6%) with testing sites both inside and outside the secure area. The most frequently offered SARS-CoV-2 test types were reverse transcription polymerase chain reaction (RT-PCR) or other NAAT (*N* = 28), followed by antigen tests (*N* = 15), and antibody tests (*N* = 8). Thirteen airports offered both RT-PCR/NAAT and antigen testing (Fig. 2).

Information on when testing first began was provided for 26 (68%) airports. Most sites began testing in the fall of 2020: five in October, seven in November, and nine in December. A few airport sites (*N* = 5) began testing as early as spring or summer of 2020.

Information on testing providers was obtained for 37 airports. Most testing providers were private companies or universities (*N* = 31; 84%). Several testing sites were run by health departments (*N* = 5; 14%) or the U.S. military (*N* = 1; 3%).

### Testing at Denver International Airport

On December 16, 2020, Denver International Airport’s testing site began offering both rapid NAAT and lab-based RT-PCR tests. The testing site is located in the secure area and open to travelers or employees with access to that area. From December 21, 2020 through March 31, 2021, 54 positive results for SARS-CoV-2 were identified out of 5724 tests conducted at the airport for a total positivity of < 1%. Of the 54 persons who tested positive at Denver International Airport, 15 were departing on imminent flights that required immediate public health action to prevent travel.

**Fig. 3:**
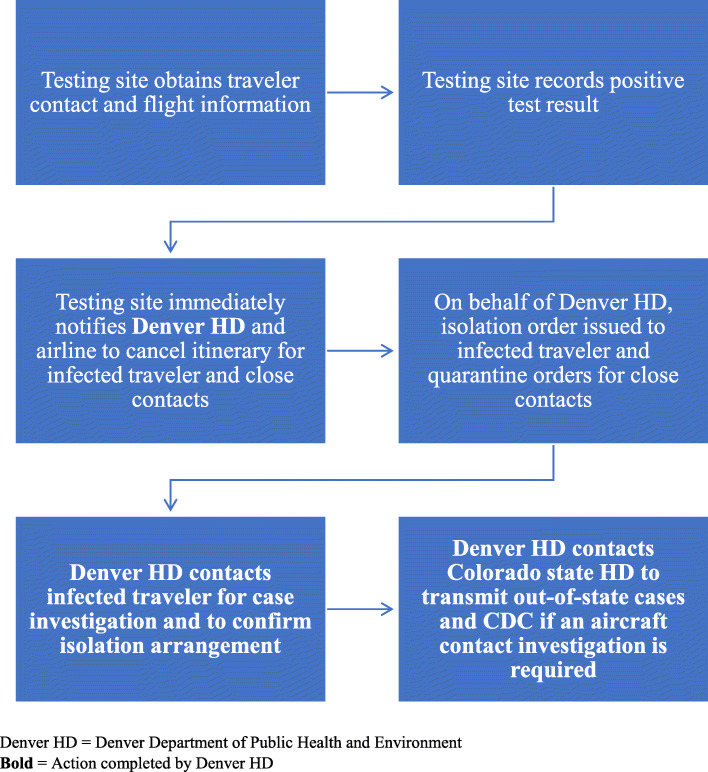
Consequence management process at Denver International Airport for travelers, December 2020–March 2021

To prevent travel of persons who test positive for SARS-CoV-2 and their travel companions, the Denver HD imposed a public health order requiring the airport’s testing site to: a) obtain accurate contact, travel, and flight information from the customer prior to testing; b) obtain plans for isolation during the infectious period if a positive result is obtained; c) immediately report positive test results to the HD; and d) notify airlines to cancel upcoming flights of travelers who test positive and their traveling close contacts (Fig. 3). Per the order, the Denver HD requires the testing site to serve the HD’s isolation orders to travelers who test positive and quarantine orders to their traveling close contacts. These travelers are responsible for securing appropriate transportation and lodging for the duration of their infectious period. Individuals who reside in Colorado cancelled or deferred their travel plans and returned to their residence to isolate after testing positive. People who tested positive who reside outside of Colorado either returned to their Colorado accommodations or in some cases rented a vehicle and drove back to their home state while agreeing to adhere to the Denver HD isolation order restricting close contact with other people. Denver HD requires the testing site to upload the isolation order to the testing site’s patient portal on the day of testing. The testing site and persons under isolation or quarantine orders are subject to penalties for failure to comply with the orders.

All 15 travelers with imminent flights who tested positive at Denver International Airport during December 21, 2020 to March 31, 2021 and their travel companions were managed under Denver’s public health orders. Because the orders specify that the testing site must immediately report cases and notify airlines to cancel upcoming flight itineraries, the Denver HD did not require use of the federal DNB list to restrict travel.

## Discussion

As of December 2020, approximately half of the surveyed US airports had COVID-19 testing sites; several more planned to start offering testing. While most sites offered testing for travelers, at some airports the testing site was open to the public in general and not exclusively to travelers, and some were only available to airport employees. This assessment was completed before many countries including the United States began enforcing testing requirements [8], and thus the availability of airport-based testing has likely increased.

The availability and large-scale adoption of testing for a communicable disease among travelers at airports is unprecedented. Border entry and exit screening operations for communicable diseases have been conducted in many countries for COVID-19 [9] and for other diseases with varying success [10–13]. However, use of onsite diagnostic testing for transiting persons at airports has rarely been adopted as a strategy to minimize transmission during travel. The increase in diagnostic testing for a notifiable disease during travel has presented new challenges for travelers, airlines, airports, and public health authorities. Persons traveling together may need to delay travel if a positive result is obtained among their travel party, which may require finding lodging for the duration of their isolation or quarantine and to get there without using public transportation. Travelers may not grasp the implications of testing positive at the airport before boarding a flight, and we found that most testing sites were private companies who may be less familiar with advising the public on consequences of testing positive prior to intended travel. Of note, the CDC currently recommends unvaccinated persons get tested 1–3 days before travel (as opposed to during travel) to help minimize transmission [4] and the aforementioned challenges.

We found that the overall positivity rate at the Denver International Airport testing site (< 1%) was substantially lower than that of the Denver community (4–6%) during the same period [14]. This may suggest that travelers are not representative of the local community and may be more likely to be asymptomatic, or that symptomatic persons may be more reticent to travel. Future studies evaluating testing site positivity across many airports should evaluate this hypothesis.

There are multiple positive attributes of Denver HD’s plan to manage persons with positive test results for SARS-CoV-2 and their close contacts at the airport testing site. Denver’s public health order for the testing site required accurate contact and flight information from customers prior to testing, which enabled immediate public health intervention upon receipt of a positive test result. Issuing isolation and quarantine orders and requiring the testing site to notify airlines immediately to cancel itineraries for upcoming flights increased adherence to public health recommendations without the need for federal public health travel restrictions. Additionally, Denver HD required any traveler seeking voluntary testing at the airport to provide plans prior to testing for appropriate housing and transportation if a positive test result was obtained, which helped minimize the logistical and financial burden on the HD to secure and provide these services.

This work is subject to at least three limitations. The results from this cross-sectional survey represented the situation in November–December 2020, and thus the proportion of airports with testing sites may have changed since the assessment. Second, we did not receive responses for 23 airports, including several large airports; therefore, our findings may be subject to response bias, if non-responding airports were more likely not to offer testing, or if airports had competing priorities due to the timing of the survey in the holiday season. Finally, our original sample selection of 100 airports may not represent all US airports since our selection criteria was focused on airports with higher passenger volume, and responding airports may be not representative of the airports in the original sample.

## Conclusion

SARS-CoV-2 testing at airports is likely to become more common as travel increases and test requirements for travel evolve. While CDC modeling indicates testing closer to the time of travel provides the greatest reduction in transmission risk while traveling [15], CDC recommends that travelers obtain testing and receive their results before arriving at the airport to avoid last-minute cancellations or itinerary changes, as well as to avoid the risks and challenges of managing infected or exposed travelers in a crowded airport environment [4]. HDs can play a key role in engaging airport testing sites to ensure people who test positive immediately before travel, and their close contact travel companions, do not travel on commercial aircraft while infected with or at-risk for SARS-CoV-2 infection.

## Data Availability

The datasets used and/or analyzed during the current study are available from the corresponding author on reasonable request.

## Abbreviations

CDC: The Centers for Disease Control and Prevention
NAAT: Nucleic acid amplification test
RT-PCR: Reverse transcription polymerase chain reaction test
DNB: Do Not Board
S/TLHD: State, local, or territorial health departments
QS: Quarantine station
HD: Health department

## Footnotes

1 See e.g., 45 C.F.R. part 46, 21 C.F.R. part 56; 42 U.S.C. §241(d); 5 U.S.C. §552a; 44 U.S.C. §3501 et seq.
